# GONNMDA: A Ordered Message Passing GNN Approach for miRNA–Disease Association Prediction

**DOI:** 10.3390/genes16040425

**Published:** 2025-04-01

**Authors:** Sihao Zeng, Shanwen Zhang, Zhen Wang, Chen Yang, Shenao Yuan

**Affiliations:** School of Electronic Information, Xijing University, Xi’an 710123, China; 18870126220@163.com (S.Z.); wangzhen4013@163.com (Z.W.); yangchen0372@163.com (C.Y.); y15294841834@163.com (S.Y.)

**Keywords:** miRNA–disease association, singular value decomposition, ordered GNN, heterogeneous graph

## Abstract

Small non-coding molecules known as microRNAs (miRNAs) play a critical role in disease diagnosis, treatment, and prognosis evaluation. Traditional wet-lab methods for validating miRNA–disease associations are often time-consuming and inefficient. With the advancement of high-throughput sequencing technologies, deep learning methods have become effective tools for uncovering potential patterns in miRNA–disease associations and revealing novel biological insights. Most of the existing approaches focus primarily on individual molecular behavior, overlooking interactions at the multi-molecular level. Conventional graph neural network (GNN) models struggle to generalize to heterogeneous graphs, and as network depth increases, node representations become indistinguishable due to over-smoothing, resulting in reduced predictive performance. GONNMDA first integrates similarity features from multiple data sources and applies noise reduction to obtain a reconstructed, comprehensive similarity representation. It then constructs heterogeneous graphs and applies a root–tree hierarchical alignment, along with an ordered gating message-passing mechanism, effectively addressing the challenges of heterogeneity and over-smoothing. Finally, a multilayer perceptron is employed to produce the final association predictions. To evaluate the effectiveness of GONNMDA, we conducted extensive experiments where the model achieved an AUC of 95.49% and an AUPR of 95.32%. The results demonstrate that GONNMDA outperforms several recent state-of-the-art methods. In addition, case studies and survival analyses on three common human cancers—breast cancer, rectal cancer, and lung cancer—further validate the effectiveness and reliability of GONNMDA in predicting miRNA–disease associations.

## 1. Introduction

Micro ribonucleic acids (miRNAs) are small RNA molecules, typically 20–24 nucleotides in length. miRNAs regulate various biological processes by targeting and modulating the transcription levels or post-transcriptional modifications of specific genes. These processes include cell proliferation and differentiation [1]. The integration of high-throughput sequencing technology with deep learning has enabled researchers to investigate miRNA expression patterns across different physiological and pathological conditions in greater depth and detail. This combination provides robust tools and data support for research on the relationship between miRNAs and diseases. Numerous studies have demonstrated that miRNAs are associated with several common human diseases, such as cancer, cardiovascular diseases, and neurological disorders. miRNAs play a critical regulatory role in the onset, progression, and development of diseases, making research on their association with diseases highly valuable in the field of biomedical science.

Studies have found that disease-sensitive regions are located in non-protein-coding areas of the genome, where non-coding RNAs play a crucial role in understanding their pathological effects. Among non-coding RNAs, miRNAs have been identified as key genetic regulators of physiological processes. Analyzing the quantitative expression levels of miRNAs provides valuable insights into human diseases. For example, miRNA interactions and regulatory functions are linked to disease progression. Chen et al. [2] found that the mature let-7 miRNA is controlled by miR-107. As let-7 acts as a tumor suppressor, the downregulation of miR-107 and inhibition of let-7 can lead to increased abundance of its target oncogenes, thereby promoting tumorigenesis. miRNAs are also critical for maintaining cellular homeostasis, regulating the cell cycle, and preventing cancerous transformations. Liu et al. [3] discovered that the loss of miR-122 regulation results in increased expression of miR-21, leading to a decrease in PDCD4 levels and the emergence of a cancerous phenotype. The upregulation of miR-21 affects cell proliferation and size, allowing for the continuous growth and survival of cancer cells. miRNAs not only function as tumor suppressors but also serve as diagnostic biomarkers. Raponi et al. [4] found that the expression of has-miR-21 in sputum provides a detection sensitivity of 70% and 100% specificity for lung cancer. In other plasma expressions, such as has-miR-210 or miR-126, sensitivity is 86% with 97% specificity. In serum, miR-200b has high accuracy in distinguishing lung cancer patients from non-cancer patients. Various miRNA families have been studied as potential diagnostic biomarkers. Calabrese [5] found that overexpression of has-miR-21, hsa-miR-200, has-miR-210, has-miR-182, and miR-183 is associated with tumor progression, while inhibition of has-miR-30 or has-miR-451 exhibits similar effects. Investigating the relationship between microRNAs (miRNAs) and human diseases provides valuable insights into the gene regulatory mechanisms and molecular networks underlying disease pathogenesis. As a critical class of non-coding RNAs (ncRNAs), miRNAs have contributed significantly to the broader advancement of non-coding transcriptomics, particularly through studies exploring their roles in health and disease. Furthermore, understanding miRNA–disease associations facilitates the identification of shared biological pathways and potential multi-disease therapeutic targets, laying a foundation for the development of cross-disease treatment strategies.

Biological wet lab experiments typically require expensive reagents, equipment, and consumables. Techniques such as real-time quantitative PCR, microarray chips, and animal studies often demand significant financial investment. High-throughput technologies enable the rapid generation of large datasets. Methods like RNA-seq, microarray technologies, and genome-wide association studies (GWAS) can simultaneously analyze thousands of genes or miRNAs. These data are integrated and analyzed using computational tools, which efficiently handle massive amounts of information, providing a broader perspective and deeper insights. Currently, there are several bioinformatics approaches to studying miRNA–disease associations, which can be broadly categorized into three types: similarity-based methods, machine-learning-based methods, and deep-learning-based methods. Similarity-based methods predict miRNA–disease associations by constructing similarity measures to assess the degree of similarity between nodes. For example, Li et al. [6] use label propagation techniques and linear neighborhood similarity (LPLNS) to predict miRNA–disease associations. Chen et al. [7] use inductive matrix completion (IMC) to predict miRNA–disease associations. This method calculates miRNA functional similarity, disease semantic similarity, and Gaussian interaction profile kernel similarity to integrate the overall similarity between miRNAs and diseases. Missing associations are then completed based on these similarities and known miRNA–disease associations. Wang et al. [8] propose a high-dimensional feature and hypergraph learning method (HFHL) for predicting miRNA–disease associations. This approach integrates miRNA functional similarity and disease semantic similarity to form high-dimensional feature vectors, constructs a hypergraph using the K-nearest neighbor method, and uses a hypergraph learning model to predict miRNA–disease associations.

Traditional machine learning methods use feature extraction techniques and classification algorithms to predict miRNA–disease associations. For example, Chen et al. [9] proposed an extreme gradient boosting machine model (EGBMMDA) for predicting miRNA–disease associations. This model trains regression trees within the gradient boosting framework to predict associations, calculating statistical measures, graph-theoretic metrics, and matrix decomposition results for each miRNA–disease pair. These results are used to form informative feature vectors, with known association pairs’ vectors used to train regression trees within the boosting framework. Wang et al. [10] developed a random-forest-based computational model (RFMDA) for predicting miRNA–disease associations. This model uses scores obtained from the random forest to predict unknown miRNA–disease associations. By integrating miRNA functional similarity, disease semantic similarity, and Gaussian interaction profile kernel similarity, it defines feature vectors representing miRNA–disease samples, with the random forest algorithm used to infer the associations between miRNAs and diseases. Ji et al. [11] introduced a network embedding learning method that uses a random forest (RF) classifier to predict potential miRNA–disease associations. This method constructs a heterogeneous information network by combining known associations between proteins, miRNAs, lncRNAs, diseases, and drugs. It uses network embedding techniques to learn behavioral information of miRNA and disease nodes, converting miRNA and disease nodes into vector representations of miRNA–disease pairs. A prediction model is then built using random forest based on the training samples. Liu et al. [12] developed a computational framework called SMALF, which uses stacked autoencoders and XGBoost to predict unknown miRNA–disease associations. The stacked autoencoders extract latent features for miRNAs and diseases from the original miRNA–disease association matrix. These latent features and similarities are cascaded to obtain feature vectors, which are then used by the XGBoost model for classification and prediction.

Deep learning methods use known associations between miRNAs, diseases, and other molecules to construct complex heterogeneous graph networks. These networks extract rich node and graph structural information to predict miRNA–disease associations. Li et al. [13] observed that prediction accuracy is affected by the sparsity of known association networks and the use of single-category features. They proposed a graph attention network (GAT) framework that simulates the complex relationships between diseases and miRNAs. Wang et al. [14] proposed a data-driven method called neural multi-category (NMC) for predicting multi-category miRNA–disease associations. The NMC encoder uses a graph neural network to learn the latent features of miRNAs and diseases separately. It then employs a graph convolutional network decoder and a neural multi-relation decoder to generate miRNA–disease association scores. He et al. [15] identified the important mediating role of genes and the issue of data sparsity. They proposed a multi-task learning miRNA–disease association (MTLMDA), which leverages miRNA–disease and gene–disease networks to improve the identification of miRNA–disease associations. Wang et al. [16] introduced a computational method called PMDAGS, PMDAGS does not rely on similarity measurements to predict potential miRNA–disease associations. Qu et al. [17] addressed the limitation of traditional matrix factorization methods, which can only extract linear features, by proposing a neural-network-based deep matrix factorization (NNDMF) method. NNDMF uses deep matrix factorization to extract nonlinear features, overcoming the shortcomings of traditional matrix factorization methods.

Despite the significant progress made by the existing methods, several challenges remain unresolved. In biological systems, molecular interactions are typically synergistic, whereas single-molecule predictions generally focus only on the behavior of individual molecules. Ignoring interactions at the multi-molecular level, such as those involving miRNA, mRNA, proteins, lncRNA, and circRNA, may lead to inaccurate or incomplete predictions. Although current GNN models integrate comprehensive graph structure information to improve miRNA–disease association predictions, they still have limitations. For instance, they struggle to generalize to heterogeneous graphs involving various biological molecules, where neighboring nodes may share different features and labels. Additionally, when these models are stacked, an over-smoothing issue arises, making it difficult to distinguish between nodes, which results in a sharp decline in performance.

Inspired by the work of Zou et al. [18], we propose a novel computational method, named GONNMDA, for predicting miRNA–disease associations. The model first integrates miRNA and disease similarity data, using singular value decomposition (SVD) to generate a reconstructed comprehensive similarity feature. Next, it constructs a comprehensive heterogeneous biological molecular graph based on known associations and captures the complex relationships between molecules through multi-level information fusion. We apply an ordered GNN model to the heterogeneous biological molecular graph, using a root–tree hierarchy to model information in various sequences. The ordered gating mechanism enables each node to extract domain-specific information and retain self-information under heterogeneous relationships, effectively alleviating the over-smoothing issue. Finally, the model combines the node feature information and similarity data to predict potential miRNA–disease associations. The key contributions of this method are as follows:

- The ordered message-passing mechanism of the ordered GNN model, guided by the root–tree hierarchy, prevents the confusion of node features during the combination stage. By modeling information in different sequences, it effectively mitigates the over-smoothing problem, where nodes become indistinguishable as the number of layers increases, thus optimizing the model’s prediction performance.
- A comprehensive biological molecular heterograph is introduced, where different types of nodes interact through various edge types. By integrating multi-level information into the heterograph, the information flow becomes more enriched.
- Multiple similarity measures are integrated, and singular value decomposition (SVD) is employed to effectively remove noise while capturing commonalities and underlying structures across different similarity types, thereby extracting more critical latent features.
- Compared to the current state-of-the-art methods, GONNMDA demonstrates outstanding performance. Case studies and survival analysis further highlight the model’s effectiveness and superiority in miRNA–disease association prediction.

## 2. Results

### 2.1. Cross Validation and Evaluation Metrics

We use cross-validation experiments to evaluate the performance of our model, which is an important tool for ensuring generalizability and preventing overfitting. The dataset is divided into several subsets, and through multiple rounds of training and validation, we ensure that each data point appears in both the training and test sets. This approach reduces the risk of overfitting or underfitting due to uneven data splits. For example, five-fold cross-validation divides the dataset into five equal subsets, or “folds”, with one fold used for validation and the remaining four used for training. The model is trained on the training set and then evaluated on the validation set. Performance metrics, such as accuracy (the proportion of correctly predicted samples), precision (the proportion of true positives among predicted positives), and recall (the proportion of correctly predicted positives among actual positives), are calculated. The F1 score is the harmonic mean of precision and recall, balancing both metrics. The calculation of these evaluation metrics is as follows:

(1) $$Accuracy=\frac{TP+FN}{TP+TN+FP+FN}$$

(2) $$Precision=\frac{TP}{TP+FP}$$

(3) $$Recall=\frac{TP}{TP+FN}$$

(4) $$F1=\frac{2\times Precision\times Recall}{Precision+Recall}$$

where true positive (TP) represents the samples that are actually positive and correctly predicted as positive. True negative (TN) represents the samples that are actually negative and correctly predicted as negative. False positive (FP) indicates that the model incorrectly predicts negative samples as positive, while false negative (FN) indicates that positive samples are incorrectly predicted as negative. We also use ROC and PR curves to evaluate the model. A higher area under the ROC curve (AUC) value, closer to 1, indicates better model performance. An area under the precision–recall curve (AUPR) near 1 indicates good precision and recall, demonstrating the model’s ability to effectively distinguish between positive and negative samples.

**Figure 1:**
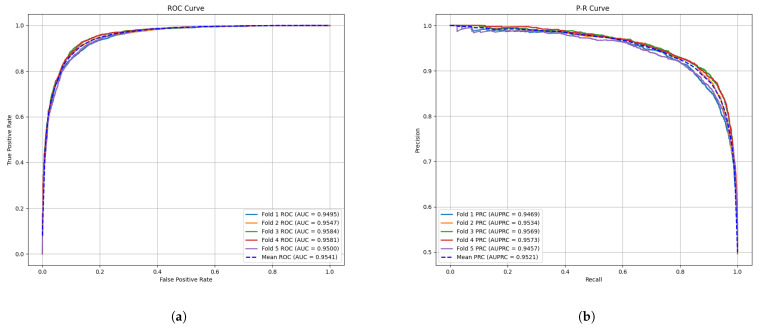
The performance of GONNMDA on 5-fold cross-validation. (**a**) ROC curves; (**b**) P-R curves.

**Figure 2:**
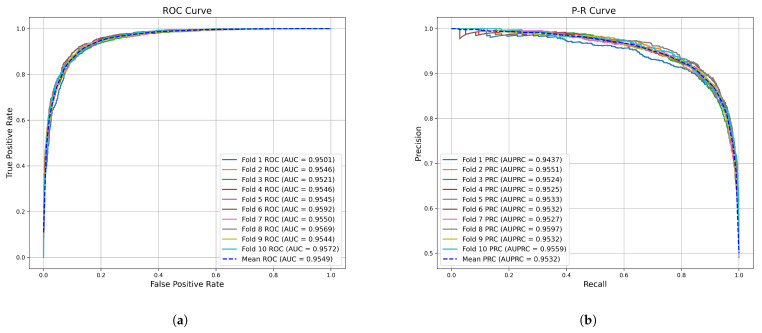
The performance of GONNMDA on 10-fold cross-validation. (**a**) ROC curves; (**b**) P-R curves.

This section presents the results of the cross-validation experiments. We conducted five-fold cross-validation on the HMDDv3.2 dataset and achieved an average accuracy of 89.01%, precision of 89.04%, recall of 88.96%, and F1 score of 89.01%. Figure 1 shows the receiver operating characteristic (ROC) curve and precision–recall (PR) curve for GONNMDA. From the figure, the AUC values are 94.95%, 95.47%, 95.84%, 95.81%, and 95.00%, with an average of 95.41% and a standard deviation of 0.0039. Additionally, from the PR curve, the AUPR values for GONNMDA are 94.69%, 95.34%, 95.69%, 95.73%, and 94.57%, with an average of 95.21% and a standard deviation of 0.0040. To further validate the model’s performance and provide a more detailed evaluation, we also conducted a ten-fold cross-validation on the HMDDv3.2 dataset. The results showed an average accuracy of 89.23%, precision of 89.30%, recall of 89.18%, and F1 score of 89.24%. As shown in Figure 2, for ten-fold cross-validation, the average AUC was 95.49% with a standard deviation of 0.0025, and the average AUPR was 95.32% with a standard deviation of 0.0013. It can be observed that the ROC curve is close to the top left corner, and the PR curve is nearly at the top right corner, demonstrating the effectiveness of the method. The performance of the ten-fold cross-validation is slightly better than that of the five-fold cross-validation, indicating that our model has strong generalization ability and can effectively adapt to patterns in the data. The similar performance between the two methods suggests that overfitting is minimal.

### 2.2. Comparative Analysis with State-of-the-Art Methods

In this section, we compare GONNMDA with state-of-the-art methods on the HMDDv3.2 dataset by implementing k-fold cross-validation. To thoroughly assess the performance of each model, we perform both five-fold and ten-fold cross-validation. For fairness, we use the best parameters provided in the original paper. Additionally, each method is evaluated through 10 experiments, and the average of the performance metrics is calculated for comparison. ROC and PR curves are also plotted.

(1) NIMCMDA [7]: This method uses potential feature representations obtained from the miRNA–disease similarity network and employs a neural inductive matrix completion model to fill the association matrix, effectively predicting potential associations between miRNAs and diseases.

(2) GCAEMDA [19]: This method leverages miRNA–miRNA similarity, disease–disease similarity, and validated miRNA–disease associations to learn embeddings for miRNA and disease nodes. It constructs miRNA-based and disease-based subnetworks, and uses a graph convolutional autoencoder to compute association scores for the two subnetworks. Finally, the scores from both subnetworks are combined to generate the miRNA–disease association score.

(3) MINIMDA [20]: This method constructs a comprehensive similarity network using multi-source miRNA information and explicitly aggregates high-order domain information to obtain embeddings for miRNA and disease. Finally, a multilayer perceptron is used to predict potential miRNA–disease associations.

(4) MTLMDA [15]: This method improves miRNA–disease association identification by incorporating a gene–disease network. It addresses data sparsity issues through the important mediating role of genes and utilizes multi-task learning to predict potential miRNA–disease associations.

(5) MVMTMDA [21]: This method leverages the known lncRNA–miRNA interaction network to predict miRNA–disease associations using a multi-view, multi-task approach, even when complete miRNA–disease information and similarity data are missing.

(6) AMHMDA [22]: This method constructs multiple similarity networks and utilizes graph convolutional networks integrated with attention mechanisms to capture rich node information from multiple perspectives. It introduces virtual nodes of known hypernodes to build a miRNA–disease heterogeneous hypergraph, and finally, the graph convolution with attention mechanism is used to predict the association scores by combining miRNA and disease features.

(7) MDformer [23]: This method integrates multiple similarity networks to obtain the embedding representations of miRNA and disease nodes. It proposes a model based on transformer architecture and meta-path instance feature encoding to generate high-quality feature embeddings for both miRNA and disease nodes. Finally, a multilayer perceptron is used to predict the miRNA–disease association scores.

(8) HGTMDA [24]: This method introduces a restart-based random walk association masking strategy to reduce noise in the dataset. After enhancing node feature representations using a graph convolutional attention mechanism, it constructs a miRNA–disease heterogeneous hypergraph. An improved GCN-Transformer encoder is then used to learn mature node embedding features, and the model is trained with the DCE loss function to predict miRNA–disease association scores.

**Figure 3:**
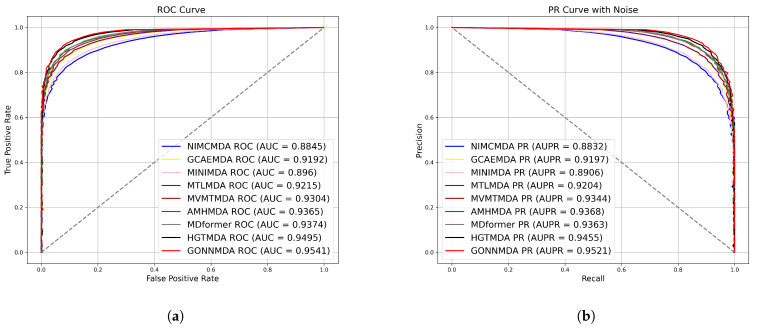
Comparison with the state-of-the-art method on 5-fold cross-validation (**a**) ROC curves; (**b**) P-R curves.

**Figure 4:**
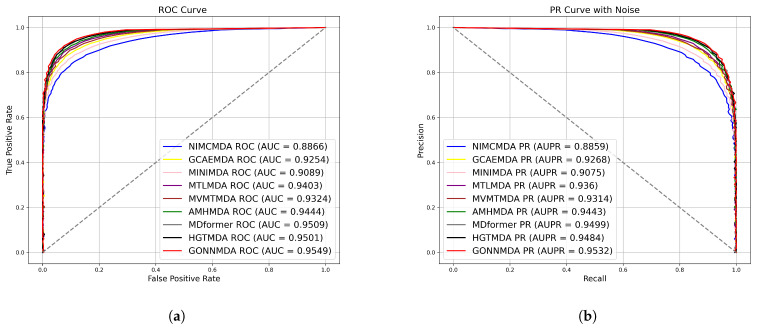
Comparison with the state-of-the-art method on 10-fold cross-validation (**a**) ROC curves; (**b**) P-R curves.

The detailed results of the comparative experiment are shown in Table 1. The results indicate that GONNMDA achieved an average AUC of 0.9541 and an average AUPR of 0.9521 in five-fold cross-validation on the HMDDv3.2 dataset. The model demonstrates high AUC and AUPR values, with a balanced precision and recall, showcasing outstanding performance. GONNMDA is slightly lower than HGTMDA by 0.0015 in recall, which can be attributed to data imbalance and model optimization for precision. In ten-fold cross-validation, GONNMDA outperformed other models with higher average AUC and AUPR values and an average accuracy of 0.8934. It is only slightly lower than the best model, MDformer, by 0.0011, proving the excellence of our proposed method. We visualized the average AUC and average AUPR of the models for comparison, as shown in Figure 3 and Figure 4. The comparison reveals the effectiveness of our proposed biomolecular heterogeneous graph and ordered graph neural network approach, with our model surpassing other state-of-the-art models.

### 2.3. Ablation Experiments

To evaluate the importance of each module in GONNMDA, we developed three variant models for performance comparison. Specifically, Model A uses only the biomolecular heterogeneous graph to obtain miRNA and disease node embeddings through ordered GNN, aiming to assess the impact of integrated similarity features on the model. Model B uses only the integrated similarity features to investigate the effect of various biomolecular nodes on the original model. Model C uses a traditional GNN without the ordered GNN to examine the effectiveness of ordered GNN in aggregating deep node information.

**Figure 5:**
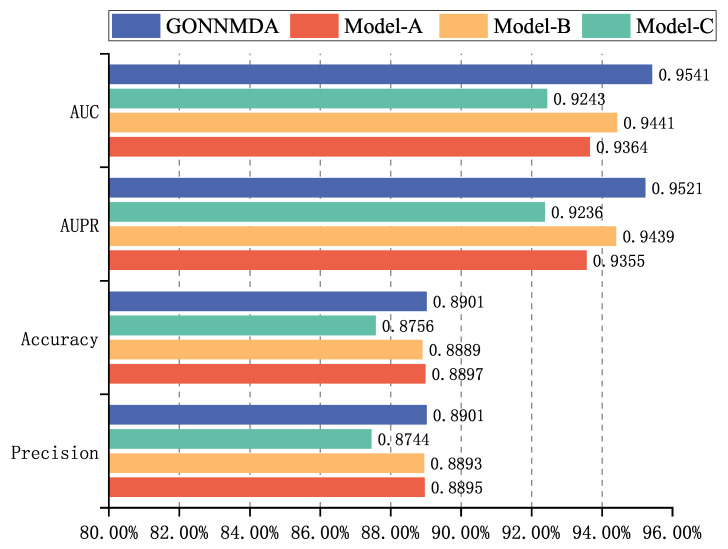
Ablation experiments with different models of GONNMDA.

The results, shown in Figure 5, reveal that the complete GONNMDA model performs the best, further demonstrating the effectiveness of integrating similarity features and using ordered GNN to generate high-quality node features. A comparison of the results from Model A and Model B shows that the importance of integrated similarity features and the structural node information from the biomolecular heterogeneous graph differs. The performance of Model C, with lower metrics than the original model, proves the effectiveness of ordered GNN in generating high-quality node embeddings.

### 2.4. Parameter Analysis

Hyperparameters have a significant impact on deep learning models, and optimal hyperparameters are crucial for model prediction performance. To analyze the influence of parameters on model predictions, we used five-fold cross-validation and grid search to find the best hyperparameters for GONNMDA. We first applied singular value decomposition (SVD) to the similarity matrix. Based on the contribution and cumulative contribution of each singular value, we selected an appropriate value of K that preserves sufficient information while minimizing the risk of overfitting and excessive computational complexity. As a result, K was set to 150. To mitigate overfitting, we applied regularization techniques by setting dropout to 0.2. The number of ordered GNN layers was set to 4, with 1024 neurons in the hidden layer of the feedforward neural network. The feature embedding dimension was set to 901, the gating vector chunk size was set to 128, and the number of layers in the input feature transformation (multilayer perceptron) was set to 2.

**Figure 6:**
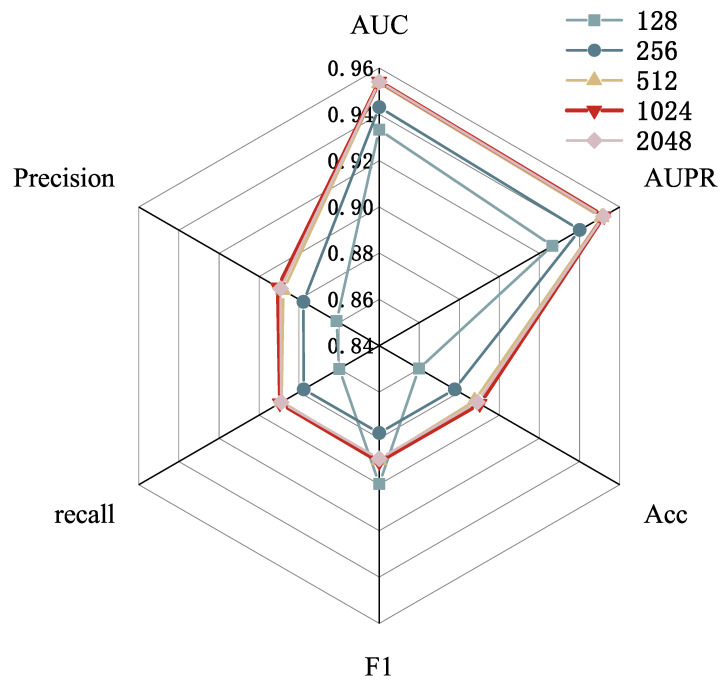
Parameter analysis for hidden layer size.

Hidden layer size. First, we adjust the size of the hidden layer, as it directly impacts the model’s capacity and expressive power. Finding an appropriate hidden layer size helps determine the overall complexity of the model. Larger hidden layers can capture more feature information but also increase computational cost and the risk of overfitting. We tested different hidden layer sizes from the set [128, 256, 512, 1024, 2048] to find the optimal configuration. As shown in Figure 6, when the hidden layer size was set to 128, the performance was relatively low, limiting the model’s expressive capability. When the hidden layer size was increased to 2048, the model’s performance did not significantly improve and even slightly decreased. The best choice was a hidden layer size of 1024, which provided optimal performance while reducing computational overhead compared to a 2048 hidden layer.

Embedding dimension. Adjusting the embedding dimension ensures that important information is not lost when input features are mapped to a lower-dimensional space. Higher embedding dimensions can enhance representational capacity, but they may also lead to redundancy. The adjustment of embedding dimensions typically has a significant impact on feature quality and model performance. We conducted experiments with various embedding dimensions, as shown in the table, where evaluation scores were recorded for dimensions [512, 600, 700, 800, 1024]. As shown in Table 2, the model’s performance improved gradually with increasing embedding dimensions, with the best performance observed at 1024. However, when the embedding dimension was increased to 2048, the model’s performance declined. Therefore, we selected 1024 as the default embedding dimension for GONNMDA.

**Figure 7:**
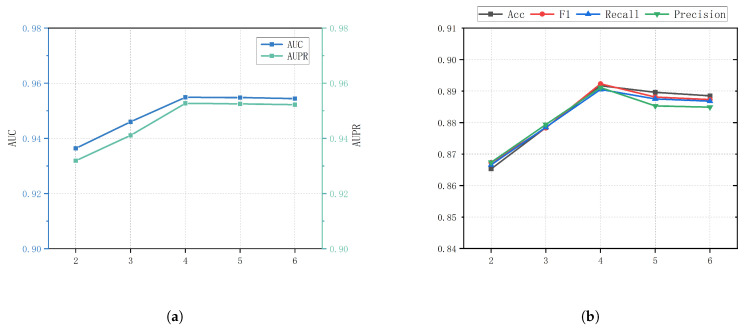
Parameter analysis for ordered GNN layers (**a**) The values of AUC and AUPR under different layers; (**b**) The values of ACC and F1 and recall and precision under different layers.

Ordered GNN layers. The number of ordered GNN layers controls the model’s depth, affecting feature extraction and the range of information propagation. With too few layers, the model cannot capture deep feature interactions, leading to suboptimal performance. On the other hand, too many layers allow the model to capture global features better but may cause excessive smoothing of the information. Figure 7 shows the performance of our model with varying numbers of ordered GNN layers. The performance steadily improved from 2 to 4 layers, with a slight decline at 5 layers, yet it remained close to the level at 4 layers. This indicates that our model effectively alleviates the over-smoothing issue. However, due to computational cost considerations, we selected 4 layers as the default number of GNN layers for the model.

**Figure 8:**
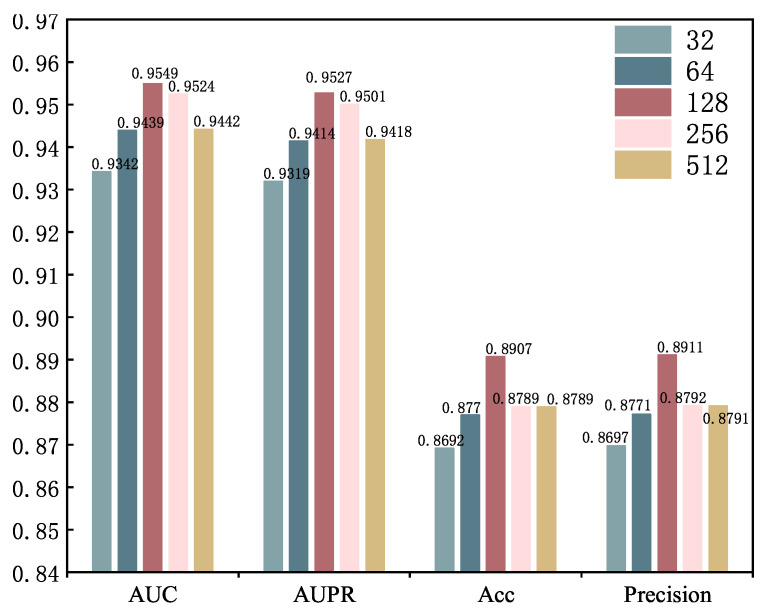
Parameter analysis for chunk size.

Chunk size. The chunk size influences the granularity of feature partitioning, and the gating vector captures detailed interactions between features by modulating them. An appropriate chunk size can stabilize the model’s performance while maintaining a high modulation capability. A smaller chunk size may lead to significant fluctuations in the gating feature modulation signal, requiring more partitioning operations and thus increasing computational cost. A larger chunk size can enhance the model’s ability to capture global features and improve computational efficiency. However, it may fail to balance the fine-grained relationships between local features, limiting its expressive power for complex feature interaction tasks. The Figure 8 shows the evaluation scores for different chunk sizes [32, 64, 128, 256, 512]. When the chunk size is 128, all performance metrics reach their highest values, indicating that this granularity is most suitable for the feature learning requirements of GONNMDA.

**Figure 9:**
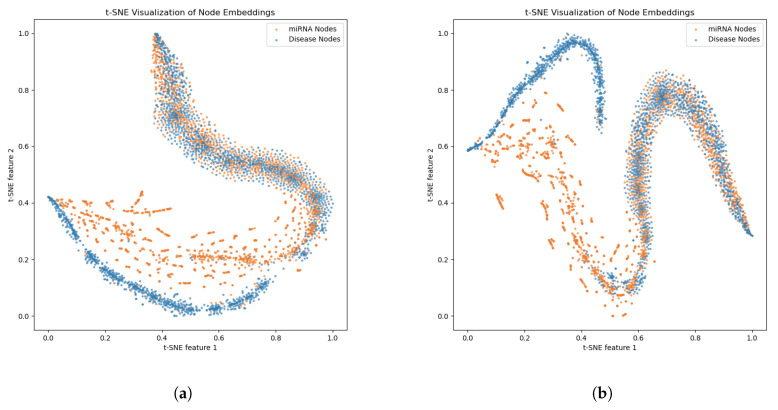

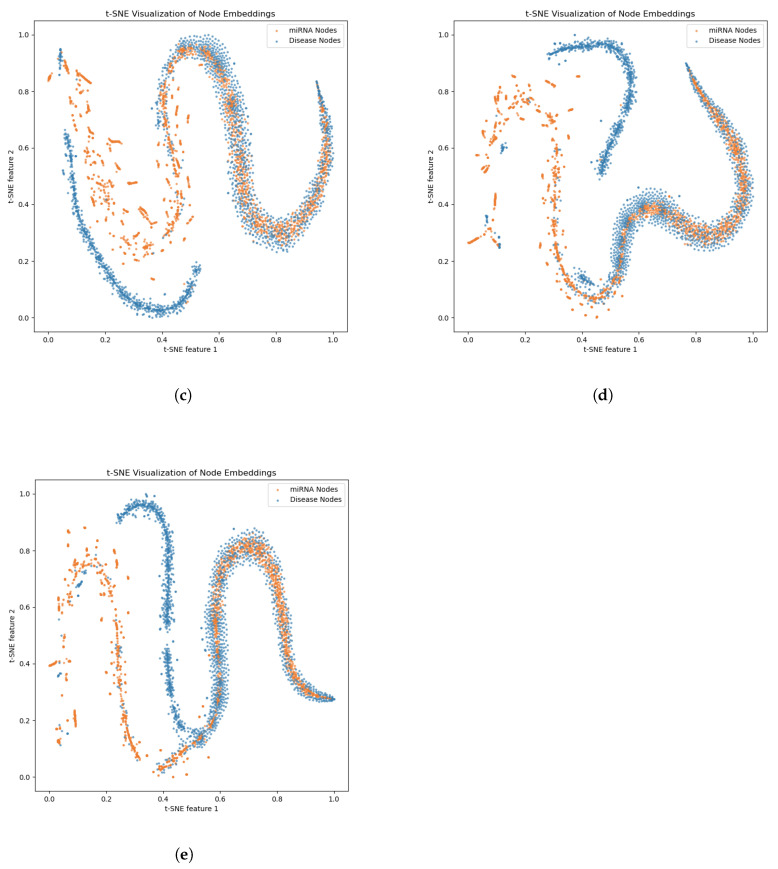
Visualization of miRNA and disease nodes embedded in different ordered GNN layers. (**a**) Layer 1; (**b**) Layer 2; (**c**) Layer 3; (**d**) Layer 4; (**e**) Layer 5.

As shown in Figure 9, the distribution of node embedding features in the t-SNE 2D space gradually changes as the number of GNN layers increases from 2 to 6. The general trend is that with fewer layers, the feature embeddings become more dispersed, causing the node distribution to become more blurred. The boundaries between miRNA and disease nodes are less clear than in higher-layer networks. With more layers, the feature embeddings become more compact, the node distribution becomes more concentrated, and the boundaries between miRNA and disease nodes are clearer. This suggests that deeper networks capture more complex structural information and offer more refined representational power. Shallow networks with 2–3 layers lack the capacity to express node features effectively, leading to a chaotic distribution of embedding features and reduced model discriminability. Deeper networks with 4–6 layers perform better in capturing miRNA and disease node features. The distribution in the embedding space is clearer, with better separation, making it more suitable for modeling complex biological molecular data. Four layers serve as a balanced compromise, retaining good separability while avoiding an overly complex model, thereby improving computational efficiency.

### 2.5. Case Studies

To validate the effectiveness of GONNMDA in predicting miRNA–disease associations in real cases, we conducted a case study involving three diseases: breast cancer, colorectal cancer, and lung cancer. We trained the model using known associations from the HMDD v3.2 database. After obtaining the embedded representations of miRNA and disease nodes, we used a multilayer perceptron to calculate the association probabilities between diseases and miRNAs. Our main goal is to identify potential associations between miRNAs and diseases. The top 30 miRNAs, ranked by their association scores, were first validated in the dbDEMC database [25]. If an miRNA was confirmed in dbDEMC, further validation in the miR2Disease database [26] was not performed, and the top 3 breast cancer miRNAs were selected for Kaplan–Meier survival analysis using clinical data from The Cancer Genome Atlas (TCGA) [27].Lung and rectal cancer survival analyses were performed in the kaplan-Meier plotter [28] database

**Figure 10:**
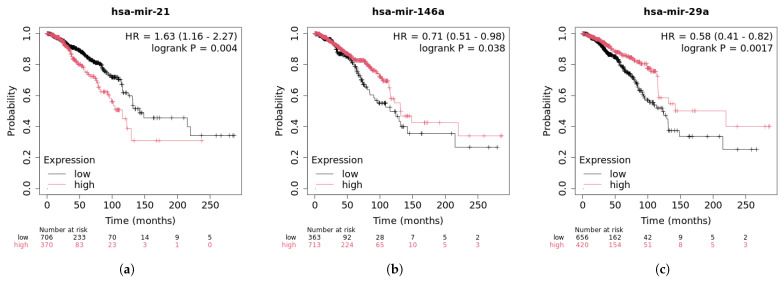
Survival analysis of top 3 predictive miRNA in breast cancer.(**a**) hsa-mir-21 survival curve; (**b**) hsa-mir-146a survival curve; (**c**) hsa-mir-29a survival curve.

Breast cancer is the most common cancer type, accounting for 23% of all cancer diagnoses among women globally [29]. It is also the leading cause of cancer-related deaths. For example, has-miR-21 promotes the proliferation of breast cancer cells by inhibiting the expression of tumor suppressor genes. The downregulation of phosphatase and tensin homolog (PTEN) leads to the activation of the PI3K/AKT signaling pathway, which, in turn, promotes cell survival and inhibits apoptosis [30]. miR-155 promotes cancer cell proliferation, survival, and metastasis by downregulating the expression of several tumor suppressor genes, including SOCS1 [31] (suppressor of cytokine signaling 1), TP53INP1 (p53-induced protein 1), and PTEN. Table 3 shows that all of the top 30 candidate miRNAs are confirmed by the database to be associated with breast cancer. Figure 10 presents the survival analysis results of the top three miRNAs.

In the case of hsa-miR-21, the low expression group exhibited longer disease-free survival and overall survival compared to the high expression group. The survival analysis showed a significant association between hsa-miR-21 expression levels and patient survival, with a *p*-value of 0.004. The hazard ratio (HR) of 1.63 further suggests that hsa-miR-21 promotes cancer progression by enhancing tumor cell proliferation, inhibiting apoptosis, increasing invasiveness, and contributing to chemotherapy resistance. In the survival analysis of hsa-miR-146a, the high expression group showed a higher survival rate than the low expression group, with a *p*-value of 0.038. This suggests that hsa-miR-146a expression may be linked to the prognosis of breast cancer patients, helping to suppress tumor progression and improve survival rates. The HR value of 0.71 indicates that patients with higher expression have a lower risk of adverse events, confirming that hsa-miR-146a can influence cell proliferation and apoptosis. High expression levels have been associated with reduced cell proliferation and increased apoptosis, further supporting its potential as a tumor suppressor. The survival curve for hsa-miR-29a shows that patients with high expression have a longer survival compared to the low expression group, with a *p*-value of 0.0017, indicating a significant correlation between hsa-miR-29a expression levels and patient survival. This further suggests that hsa-miR-29a acts mainly as a tumor suppressor in breast cancer. It inhibits tumor cell proliferation, promotes apoptosis, suppresses invasion and metastasis, modulates the tumor microenvironment, and enhances chemotherapy sensitivity, thereby inhibiting breast cancer development and malignancy. Due to its tumor-suppressing effects, hsa-miR-29a is an important therapeutic target and prognostic biomarker.

**Figure 11:**
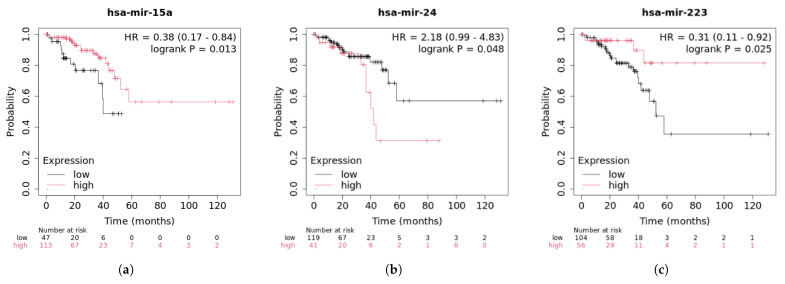
Survival analysis of top 3 predictive miRNA in rectal cancer.(**a**) hsa-mir-15a survival curve; (**b**) hsa-mir-24 survival curve; (**c**) hsa-mir-223 survival curve.

Rectal adenocarcinoma is a common type of malignant tumor in the gastrointestinal tract, accounting for 8% of global cancer incidence and mortality [32]. Research into rectal cancer focuses on the discovery of novel biomarkers, the optimization of personalized treatments, and the development of new immunotherapies and targeted therapies. For example, hsa-miR-15a inhibits the proliferation of rectal cancer cells by regulating cell-cycle-related genes. It targets and downregulates the expression of CDK6 (Cyclin-Dependent Kinase 6) and Cyclin D1 [33], which play crucial roles in the cell cycle process. hsa-miR-24 promotes cancer cell proliferation by downregulating tumor suppressor genes such as CDKN1B [34], which encodes p27-Kip1, a cell cycle inhibitor. CDKN1B typically limits cell proliferation by inhibiting the cell cycle process. As shown in Table 4, all of the top 30 miRNAs associated with colorectal cancer have been validated. Figure 11 presents the survival analysis results for the top three ranked miRNAs in rectal cancer patients.

The survival analysis of rectal cancer patients reveals that those with high expression of hsa-miR-15a have longer survival than those with low expression, and the number of low-expression patients surviving beyond 60 months sharply decreases. A *p*-value of 0.013 indicates statistical significance, while an HR of 0.38 suggests that patients with high expression of hsa-miR-15a have a 62% lower risk of experiencing adverse events compared to those with low expression. These results indicate that hsa-miR-15a, as a tumor suppressor, may provide a protective effect for rectal cancer patients, potentially prolonging survival and improving prognosis. The survival curve for hsa-miR-24 shows that a *p*-value of 0.048 indicates statistical significance, though it is close to the threshold for significance. The survival curve for the high-expression hsa-miR-24 patient group declines more steeply, indicating that these patients have a shorter survival time and a faster decline in survival rate. hsa-miR-24 may serve as a negative prognostic marker for predicting poor survival outcomes in rectal cancer patients. High expression of hsa-miR-24 is likely associated with poorer prognosis, suggesting that these patients have a higher risk of recurrence and shorter survival. The survival curve for hsa-miR-223 shows that the high-expression patient group has a higher survival rate, with a slower decline in the curve, indicating longer survival for these patients. The low-expression group, on the other hand, shows a faster decline in survival rate, indicating that low expression of hsa-miR-223 is associated with poorer survival prognosis. A *p*-value of 0.025 suggests that the survival difference between high and low expression of hsa-miR-223 is a true association. An HR of 0.3 indicates that patients with high expression of hsa-miR-223 have a 69% lower risk of experiencing adverse events (such as death or recurrence) compared to those with low expression. hsa-miR-223 has a significant protective effect in these patients, and its high expression is associated with better survival prognosis. Further research into the biological mechanisms of hsa-miR-223, including how it affects processes such as tumor cell proliferation, apoptosis, and invasion, could provide a foundation for developing new therapeutic strategies.

**Figure 12:**
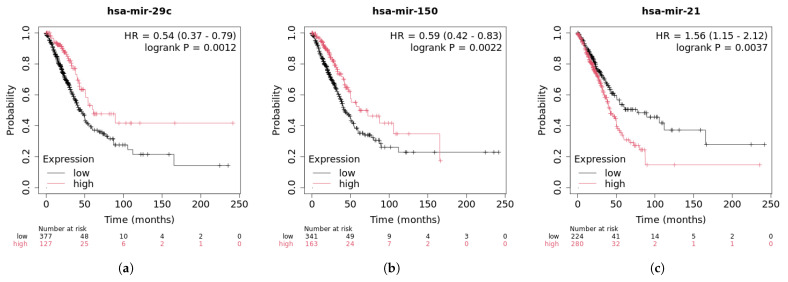
Survival analysis of top 3 predictive miRNA in lung cancer.(**a**) hsa-mir-29c survival curve; (**b**) hsa-mir-150 survival curve; (**c**) hsa-mir-21 survival curve.

Lung cancer is one of the most common cancers and ranks second globally in terms of new cases [35]. It is also the leading cause of cancer-related deaths worldwide. MiR-17-92 is upregulated in the early stages of lung cancer but decreases as the disease progresses. Inhibition of hsa-miR-17 expression can suppress lung cancer cell proliferation and induce apoptosis. Hsa-miR-1 can inhibit the expression of oncogenes, thereby affecting lung cancer cell proliferation and migration. A549 cells with ectopic expression of miR-1 can activate caspases-3 and caspases-7 in response to doxorubicin, triggering apoptosis [36]. As shown in Table 5, the top 30 predicted miRNAs associated with lung cancer have been validated by the database. Figure 12 presents the survival analysis results of the top three miRNAs for lung cancer patients.

The survival analysis of lung cancer patients reveals that high expression of hsa-miR-29c is significantly associated with a reduced risk of adverse events. This correlation is statistically significant. This suggests that hsa-miR-29c may act as a tumor suppressor, playing a protective role in lung cancer, potentially extending patient survival and improving prognosis. The hazard ratio (HR) for hsa-miR-150 is 0.59, indicating that patients with high expression of hsa-miR-150 have a 59% lower risk of death or relapse during follow-up compared to those with low expression. The *p*-value is less than 0.05 and much smaller than 0.01, indicating that the survival difference between high and low expression of hsa-miR-150 is highly statistically significant. This suggests that the difference is likely due to the expression level of hsa-miR-150, which may serve as a protective factor associated with a lower risk of death or relapse, with high expression potentially correlating with longer survival. Therefore, hsa-miR-150 may serve as a valuable prognostic biomarker for rectal cancer patients. The hazard ratio (HR) for hsa-miR-21 is 1.56, meaning that patients with high expression of hsa-miR-21 face a 1.56 times higher risk during their survival period compared to those with low expression. The *p*-value is less than 0.05, indicating statistical significance, which means that the survival difference between high and low expression groups is not coincidental but likely due to differences in gene expression levels. Hsa-miR-21 may act as an oncogene, potentially linked to tumor progression, metastasis, or resistance to treatment.

To compare the role of specific miRNAs in disease, we selected miR-223-5p as a representative example. miR-223-5p is a microRNA with important regulatory functions across various disease states. It plays a key role in several biological processes, including inflammation, cell proliferation, differentiation, and immune regulation. In cancer-related studies, the role of miR-223-5p varies by tumor type. For instance, it is typically downregulated in gastric cancer, hepatocellular carcinoma, and breast cancer, where it acts as a tumor suppressor by targeting multiple oncogenes and inhibiting tumor cell migration and invasion. Conversely, in certain types of leukemia, it is upregulated and may be associated with disease progression. In cardiovascular diseases, miR-223-5p is generally considered cardioprotective. Its upregulation can reduce cardiomyocyte apoptosis and inflammation, showing potential diagnostic and prognostic value in conditions such as myocardial infarction and atherosclerosis. Overall, the function of miR-223-5p exhibits strong tissue- and pathology-specific characteristics across different diseases. It holds considerable promise for applications in disease diagnosis, biomarker development, and the identification of therapeutic targets.

## 3. Materials and Methods

### 3.1. Dataset

In this study, we obtained the HMDDv3.2 from The Human microRNA Disease Database [37], which includes 35,547 miRNA–disease associations involving 901 miRNAs and 877 diseases. After filtering, we identified 15,186 unique experimentally validated associations and selected an equal number of non-associated pairs through random negative sampling as negative samples. Furthermore, to construct the biological entity network, we used additional biological molecules curated by Zou et al. [18], including 3348 proteins, 3024 mRNAs, 2633 lncRNAs, 1319 drugs, 421 circRNAs, and 100 microorganisms. Additionally, we organized 421 circRNA–disease associations from circRNADisease [38], 1378 circRNA–miRNA associations from CircInteractome [39] and circBase [40], and 3416 mRNA–disease and lncRNA–disease associations from RNADisease [41]. We also curated 175 microorganism–disease associations from HMDAD [42], 17,414 drug–disease relationships, 11,396 drug–protein interactions, and 269 miRNA–drug associations from DrugBank [43]. We retrieved 800 drug–microbe relationships from MagMD [44], 3915 mRNA–drug associations from PharmGKB [45], and 874 lncRNA–disease associations from LncRNAdisease [46]. From starBase [47], we obtained 8634 lncRNA–miRNA associations, 525 lncRNA–mRNA interactions, and 5186 miRNA–mRNA relationships. We also filtered 5115 lncRNA–protein interactions from NPInter [48]. Additionally, we curated 2042 miRNA–protein relationships from STRING [49] and 3012 mRNA–protein interactions from NCBI [50].

### 3.2. GONNMDA

**Figure 13:**
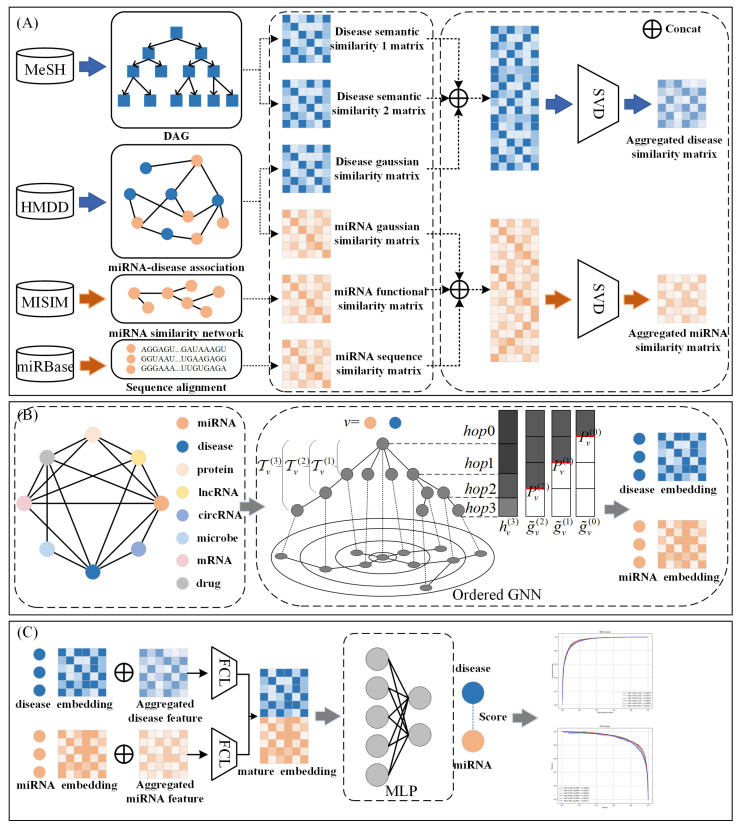
The framework of GONNMDA.(**A**) Reconstructed similarity feature; (**B**) Ordered GNN; (**C**) Multilayer perceptron.

In this section, we introduce the basic framework of the proposed GONNMDA. Specifically, (1) integrating miRNA similarity features and disease similarity features, (2) applying singular value decomposition (SVD) to extract critical latent information and obtain reconstructed comprehensive similarity features, (3) constructing a biomolecular heterogeneous graph and using an ordered GNN to learn rich multi-level information for miRNA node and disease node features, and (4) finally, using a multilayer perceptron to predict potential miRNA–disease associations. Figure 13 illustrates the overall workflow of our method.

#### 3.2.1. Disease Semantic Similarity

Based on the descriptors obtained from MeSH [51], the relationships between diseases can be represented as a directed acyclic graph (DAG), which includes the disease and all of its ancestor disease nodes. A disease DAG is denoted as $DAG_{d}=\left(d,T_{d},E_{d}\right)$, where *d* represents the disease, $T_{d}$ is the set containing disease *d* and all its ancestor nodes, and $E_{d}$ is the set of corresponding edges. The semantic value $D_{d}\left(t\right)$ of disease *d* is calculated based on the DAG structure. For each disease node *t*, its semantic value depends on its relationships with its child nodes, as expressed in the following formula:

(5) $$\left\{\begin{array}{cc} D_{d}\left(d\right)=1 & t=d\\ D_{d}\left(t\right)=\max(\lambda \cdot D_{d}\left(t^{\prime }\right),t^{\prime }\in children\;of\;t)) & t\neq d \end{array}\right.$$

where λ is the semantic contribution factor of the edge, and $D_{d}\left(t^{\prime }\right)$ represents the semantic value of the subset of disease nodes. Based on the DAG of each disease, the overall semantic value Ds1(d) of the disease can be computed as the sum of the semantic contributions of the disease and all its ancestor nodes:

(6) $$Ds1\left(d\right)=\sum_{t\in T_{d}}D_{d}\left(t\right)$$

The semantic similarity between any two diseases is calculated based on the intersection of their semantic values in their respective DAGs. According to Equations (1) and (2), the disease semantic similarity matrix DSS1 is obtained, as shown in the following formula:

(7) $$DSS1\left(d_{i},d_{j}\right)=\frac{\sum_{t\in T_{d_{i}}\cap T_{d_{j}}}\left(D_{d_{i}}\left(t\right)+D_{d_{j}}\left(t\right)\right)}{Ds1\left(d_{i}\right)+Ds1\left(d_{j}\right)}$$

To ensure semantic completeness, different semantic contribution values are assigned within the same layer using various methods to complement the specific functional information of the diseases. The second semantic similarity calculation is as follows:

(8) $$Ds2\left(d\right)=\sum_{t\in T_{d}}-\log\left(\frac{\left|G_{d}\left(t\right)\right|}{\left|N_{DAG}\right|}\right),$$

where $\left|G_{d}\left(t\right)\right|$ represents the number of times disease node *t* appears across all DAGs, and $\left|N_{DAG}\right|$ represents the total number of DAGs. The second semantic similarity matrix DSS2 can then be calculated as follows:

(9) $$DSS2\left(d_{i},d_{j}\right)=\frac{\sum_{t\in T_{d_{i}}\cap T_{d_{j}}}\left(D_{d_{i}}\left(t\right)+D_{d_{j}}\left(t\right)\right)}{Ds2\left(d_{i}\right)+Ds2\left(d_{j}\right)},$$

Gaussian kernel similarity for diseases is a nonlinear method for computing the similarity based on disease feature vectors. This approach maps the disease features into a high-dimensional space to calculate their similarity. The kernel similarity between diseases $d_{i}$ and $d_{j}$ is calculated as follows:

(10) $$DGS\left(d_{i},d_{j}\right)=\exp\left(-\sigma {∥IP\left(d_{i}\right)-IP\left(d_{j}\right)∥}^{2}\right),$$

where σ represents the kernel bandwidth, which controls the rate at which similarity decays, and $∥IP\left(d_{i}\right)-IP\left(d_{j}\right)∥$ denotes the Euclidean distance between diseases $d_{i}$ and $d_{j}$. The kernel bandwidth σ is calculated as follows:

(11) $$\sigma =\frac{1}{\frac{1}{N_{d}}\sum_{k=1}^{N_{d}}{∥IP(d_{k})∥}^{2}},$$

where $∥IP(d_{k})∥$ represents the length of the binary vector, $N_{d}$ represents the number of diseases.

#### 3.2.2. MiRNA Similarity

The miRNA functional similarity algorithm is primarily based on miRNA–disease association data and disease semantic similarity. The method first uses the precomputed disease semantic similarity, then integrates miRNA–disease association data with the disease similarity matrix, and calculates miRNA functional similarity using cosine similarity. Based on the information from the MISIM v2.0 database provided by Li et al. [52], we have computed the miRNA functional similarity matrix used in this study. Based on the upregulation and downregulation relationships between miRNAs and diseases, the overall semantic value of a disease can be represented as $\Delta D_{s}\left(d\right)$, calculated as follows:

(12) $$\Delta Ds(d)={\left(-1\right)}^{k}\sum_{t\in T_{d}}D_{d}\left(t\right),$$

where *k* represents the upregulation and downregulation relationship of miRNAs in diseases. When k=0, it indicates upregulation, and when k=1, it indicates downregulation. Based on Equation (4), this relationship can be quantified as a semantic feature vector, as shown below:

(13) $$mf_{i}=\{\begin{array}{c} \Delta Ds\left(d_{i1}\right),\Delta Ds\left(d_{i2}\right),\cdots ,\Delta Ds\left(d_{in}\right) \end{array}\},$$

where $d_{in}$ represents the n-th disease associated with the *i*-th miRNA. The semantic similarity between diseases associated with any two miRNAs can be calculated as follows:

(14) $$mdf\left(m_{i},m_{j}\right)=\left\{\begin{array}{c} DSS\left(d_{i1},d_{j1}\right),DSS\left(d_{i2},d_{j2}\right),\cdots ,DSS\left(d_{in},d_{jm}\right) \end{array}\right\},$$

The improved semantic features of diseases associated with miRNA are as follows:

(15) $$fm_{i}=\left\{\begin{array}{c} mf_{i},mdf(m_{i},m_{j}) \end{array}\right\},$$

Using the disease semantic similarity features calculated in Equation (6), the miRNA functional similarity matrix is computed as follows:

(16) $$MFS\left(m_{i},m_{j}\right)=\frac{fm_{i},fm_{j}^{T}}{∥fm_{i}∥\cdot ∥fm_{j}∥},$$

miRNA Sequence Similarity: miRNAs typically consist of 20–23 nucleotides, making their sequence lengths similar. The Needleman–Wunsch algorithm [53] is used to compare the similarity of miRNA sequences. We obtained miRNA sequence data from the public miRBase database. To integrate with other similarity metrics, the calculated sequence similarity is normalized. The computation is as follows:

(17) $$MQS\left(m_{i},m_{j}\right)=\left\{\begin{array}{cc} 1 & i=j\\ \frac{score\left(m_{i},m_{j}\right)-score_{\min}}{score_{\max}-score_{\min}} & i\neq j \end{array}\right.,$$

where $score(m_{i},m_{j})$ represents the matching score between any two miRNAs, $score_{\max}$ denotes the maximum score, and $score_{\min}$ denotes the minimum score.

Similar to the Gaussian kernel similarity for diseases, the Gaussian kernel similarity for miRNAs calculates similarity by mapping miRNA features to a high-dimensional space. The kernel similarity between miRNAs $m_{i}$ and $m_{j}$ is computed as follows:

(18) $$MGS\left(m_{i},m_{j}\right)=\exp\left(-\sigma {∥IP\left(m_{i}\right)-IP\left(m_{j}\right)∥}^{2}\right),$$

where σ represents the kernel bandwidth, which controls the rate of similarity decay, and $∥IP\left(m_{i}\right)-IP\left(m_{j}\right)∥$ represents the Euclidean distance between diseases $m_{i}$ and $m_{j}$. The kernel bandwidth σ is calculated as follows:

(19) $$\sigma =\frac{1}{\frac{1}{N_{m}}\sum_{k=1}^{Nm}{∥IP(m_{k})∥}^{2}},$$

where $∥IP(m_{k})∥$ represents the binary vector, and $N_{m}$ represents the number of diseases.

#### 3.2.3. Reconstructed Comprehensive Similarity Features

miRNA sequence similarity is primarily based on the nucleotide sequences of miRNAs, which helps identify evolutionary relationships and conservation among miRNAs. However, it does not directly reflect the association between miRNAs and diseases. miRNA functional similarity, based on miRNA–disease association data, may introduce noise due to incomplete data. The Gaussian kernel similarity of miRNAs can capture complex similarity patterns, but it is dependent on the features used for representation. Different similarity measures capture various aspects of miRNAs, and integrating them helps obtain a comprehensive set of feature information. The similarity matrices obtained from Equations (8)–(10) are concatenated. The concatenated feature matrix is then subjected to SVD decomposition. By truncating the SVD, noise is removed and important information is extracted. The reconstructed miRNA comprehensive similarity matrix is calculated as follows:

(20) $$Sm_{combined}=\left[\begin{array}{c} MFS\\ MQS\\ MGS \end{array}\right]=U\Sigma V^{T},$$

(21) $$Sm_{reconstructed}=U_{k}\Sigma_{k}V_{k}^{T},$$

where $U_{k}\in R^{3m\times k}$ represents the left singular vectors, $\Sigma_{k}\in R^{k\times m}$ denotes the diagonal matrix of singular values, $V_{k}^{T}\in R^{m\times k}$ indicates the right singular vectors, and *k* refers to the number of singular values.

After further adjustment and optimization of the matrix through a linear layer, we obtain:

(22) $$H_{aggmiR}=Linear\left(Sm_{reconstructed}\right),$$

Disease semantic similarity offers deep biological or functional insights, while disease Gaussian kernel similarity, based on the distance and similarity between features, captures finer-grained information through the relationships between numerical features. Integrating both methods provides a more comprehensive assessment of disease similarity. After concatenating the similarity matrices obtained from Equations (3), (5), and (6), singular value decomposition (SVD) is applied to reconstruct the comprehensive disease similarity, calculated as follows:

(23) $$Sd_{combined}=\left[\begin{array}{c} DSS1\\ DSS2\\ DGS \end{array}\right]=U\Sigma V^{T},$$

(24) $$Sd_{reconstructed}=U_{k}\Sigma_{k}V_{k}^{T},$$

(25) $$H_{aggdis}=Linear\left(Sd_{reconstructed}\right),$$

#### 3.2.4. Heterogeneous Biological Molecular Graph

We use the curated biological molecules as nodes in the heterogeneous graph and the relationships between different biological molecules as the edges of the graph. Let the biological heterogeneous network be represented by $G_{Bio}=\left(V,E,T_{V},T_{E}\right)$, where *V* denotes the set of nodes, *E* denotes the set of edges, and $T_{V}$ and $T_{E}$ represent the sets of node and edge types, respectively. The set *V* contains miRNA, diseases, drugs, mRNA, proteins, lncRNA, microorganisms, and circRNA, each assigned a unique label from the set [0,1,2,3,4,5,6,7]. All different types of nodes are mapped to initial node vectors. The set *E* establishes edge indices using a dictionary containing pairs of tuples, with each edge relationship represented by a unique number, including 16 different types of edge relationships. The biological heterogeneous graph includes multiple types of nodes and various edge relationships between them. An ordered message-passing strategy helps the model handle different nodes and edges in stages during propagation, thereby better reflecting the complex interactions among various node types.

#### 3.2.5. Ordered GNN

In this section, we will provide a detailed description of the steps involved in constructing the ordered GNN. The ordered GNN consists of three main modules: (1) root–tree nesting structure; (2) split point operation; and (3) soft gating mechanism.

The root–tree nesting structure begins by constructing a rooted-tree hierarchy for each central node. This hierarchy organizes the neighboring nodes in layers, starting from the central node, with each level representing the neighbors at a certain “hop” distance. The first layer consists of the node’s direct neighbors (one-hop neighbors), the second layer includes the neighbors of the one-hop neighbors (two-hop neighbors), and so on. The model then arranges the neurons in an ordered manner and aligns this rooted-tree structure with the ordered neurons of the nodes. This ensures that one-hop information is passed only to specific neuron blocks in the node’s representation, while two-hop information is transmitted to a different set of neurons. This alignment ensures the sequential flow of information across the hierarchy. The nested root–tree structure can be represented as

(26) $$T_{\nu }^{\left(0\right)}\subseteq T_{\nu }^{\left(1\right)}\subseteq \cdots \subseteq T_{\nu }^{\left(k-1\right)}\subseteq T_{\nu }^{\left(k\right)}\subseteq \cdots \subseteq T_{\nu }^{\left(K\right)},$$

where $T_{\nu }^{\left(0\right)}$ represents the initial information neurons of the node, and $T_{\nu }^{\left(k-1\right)}$ is a subset of $T_{\nu }^{\left(k\right)}$.

The split point divides the node embedding representation $h_{v}$ into multiple parts, each corresponding to the neighbor information at different levels of the rooted-tree hierarchy. The information from the k-th hop neighbors is mapped to the p(k)-th group of neurons in the node embedding. For different levels of the tree, the number of neurons used to encode neighbor information varies. The ordered neuron allocation ensures that the messages from the k-hop neighbors only affect the specific part of the node embedding. The node embedding representation h is split by the split point as follows:

(27) $$P_{\nu }^{\left(0\right)}\leq P_{\nu }^{\left(1\right)}\leq \cdots \leq P_{\nu }^{\left(k-1\right)}\leq P_{\nu }^{\left(k\right)}\leq \cdots \leq P_{\nu }^{\left(K\right)},$$

To ensure alignment, the ordered GNN introduces a soft gating mechanism. The gating vector $g_{\nu }^{\left(k\right)}$ controls which neurons retain messages from the previous layer and which neurons receive messages from the current layer. This gating vector is adjusted according to the hierarchy of the rooted tree, ensuring orderly message passing. A D-dimensional binary gating vector is used to control the split point $P_{\nu }^{\left(k\right)}$, which divides the ordered node embedding into two parts. The first part, from indices $\left[0,P_{\nu }^{\left(k\right)}-1\right]$, consists of 1’s, indicating that these neurons retain the output from the previous layer. The second part, from indices $\left[P_{\nu }^{\left(k\right)},D\right]$, consists of 0’s, indicating that these neurons allow new neighbor information to pass through. The node features from the (k − 1)-th layer, $h_{\nu }^{\left(k-1\right)}$ are used as input, along with contextual information $m_{\nu }^{\left(k\right)}$, during the message update phase at the k-th layer, calculated as follows:

(28) $$h_{\nu }^{\left(k\right)}=g_{\nu }^{\left(k\right)}*h_{\nu }^{\left(k-1\right)}+\left(1-g_{\nu }^{\left(k\right)}\right)*m_{\nu }^{\left(k\right)},$$

where ∗ denotes element-wise multiplication.

Due to the discrete binary nature and the clear boundary at $P_{\nu }^{\left(k\right)}$, the model becomes non-differentiable. To maintain the differentiability of the entire ordered GNN, we use the expected value of the predicted gate to perform a “softening” operation. In this step, we concatenate the output vector $h_{\nu }^{\left(k-1\right)}$ from the previous layer with the neighbor context vector $m_{\nu }^{\left(k-1\right)}$, apply a linear layer, and then perform a softmax operation to obtain the expected value. The expected values are accumulated to produce the gate expectation vector, calculated as follows:

(29) $${\hat{g}}_{\nu }^{\left(k\right)}=cumsum_{\leftarrow }\left\{f_{\xi }^{\left(k\right)}\left(h_{\nu }^{\left(k-1\right)},m_{\nu }^{\left(k\right)}\right)\right\},$$

(30) $$f_{\xi }^{\left(k\right)}=softmax\left(W^{\left(k\right)}\left[h_{\nu }^{\left(k-1\right)};m_{\nu }^{\left(k\right)}\right]+b^{\left(k\right)}\right)\;,$$

where $cumsum_{\leftarrow }\left(•\right)$ denotes the cumulative sum operation from right to left, $f_{\xi }^{\left(k\right)}$ represents the function for merging two vectors, $W^{\left(k\right)}$ refers to the learnable parameters, $b^{\left(k\right)}$ is the bias term, and $\left[h_{\nu }^{\left(k-1\right)};m_{\nu }^{\left(k\right)}\right]$ indicates the concatenation of two vectors. To ensure that the predicted splitting point aligns with the division in Equation (13) without disrupting the alignment between the rooted tree and node embeddings, we perform bitwise operations on the gate expectation vector. The newly calculated SOFTOR gate is as follows:

(31) $${\tilde{g}}_{\nu }^{\left(k\right)}={\tilde{g}}_{\nu }^{\left(k-1\right)}+\left(1-{\tilde{g}}_{\nu }^{\left(k\right)}\right)*{\hat{g}}_{\nu }^{\left(k\right)},$$

The updated embedding vector for the k-th layer that we ultimately obtain is

(32) $$h_{\nu }^{\left(k\right)}={\tilde{g}}_{\nu }^{\left(k\right)}*h_{\nu }^{\left(k-1\right)}+\left(1-{\tilde{g}}_{\nu }^{\left(k\right)}\right)*m_{\nu }^{\left(k\right)},$$

Two rooted-tree structures are constructed with the miRNA nodes and disease nodes as the centers in the biological molecular heterogeneous graph. From Equation (17), the representations of all miRNA and disease nodes can be obtained.

## 4. Conclusions

In this study, we propose a new computational method called GONNMDA for predicting potential miRNA–disease associations. GONNMDA integrates multi-source similarity features and utilizes graph structural features from heterogeneous graphs composed of various biomolecule relationships to generate high-quality, mature node representations for prediction. To handle the heterogeneous graph with multiple biomolecule associations, an ordered graph neural network (GNN) is introduced. This model uncovers deeper graph relationships through the rooted-tree alignment structure of the ordered GNN, effectively alleviating the over-smoothing problem in multi-node learning of biological heterogeneous graphs.

To evaluate the performance of our proposed model, we conducted a series of experiments, including five-fold cross-validation and ablation studies, which confirmed the robustness and reliability of GONNMDA. Furthermore, we conducted detailed case studies of the model’s application to breast cancer, rectal cancer, and lung cancer, followed by survival analysis using clinical data. The results demonstrate that our model accurately predicts miRNA–disease associations. These findings validate the feasibility of our approach and provide new insights and tools for uncovering the potential mechanisms underlying miRNA-related diseases.

## Figures and Tables

**Table 1:** Comparison with the state-of-the-art method on HMDD v3.2 dataset.

	Method	AUC (%)	AUPR (%)	Accuracy (%)	Precision (%)	Recall (%)	F1-Score (%)
5-fold	NIMCMDA	88.45	88.32	81.28	80.76	81.22	81.48
	GCAEMDA	91.92	91.97	84.15	85.18	88.87	84.85
	MINIMDA	89.60	89.06	85.54	85.43	86.73	85.18
	MTLMDA	92.15	92.04	84.99	83.37	88.89	85.45
	MVMTMDA	93.04	93.44	84.83	84.81	85.29	85.13
	AMHMDA	93.65	93.68	86.08	86.33	84.89	84.55
	MDformer	93.74	93.63	87.84	89.00	88.19	87.66
	HGTMDA	94.95	94.55	88.95	88.90	**89.11**	88.93
	GONNMDA	**95.41**	**95.21**	**89.01**	**89.04**	88.96	**89.01**
10-fold	NIMCMDA	88.66	88.59	81.45	80.93	81.50	82.01
	GCAEMDA	92.54	92.68	85.43	86.12	89.42	86.03
	MINIMDA	90.89	90.75	87.76	87.77	88.46	86.88
	MTLMDA	94.03	93.60	87.16	88.13	89.56	87.13
	MVMTMDA	93.24	93.14	85.46	86.17	83.64	86.66
	AMHMDA	94.44	94.43	86.78	88.41	89.00	87.10
	MDformer	95.09	94.99	**89.34**	88.97	88.49	88.95
	HGTMDA	95.01	94.84	88.95	88.89	89.12	89.20
	GONNMDA	**95.49**	**95.32**	89.23	**89.30**	**89.18**	**89.24**

Bold indicates best performance.

**Table 2:** Parameter analysis for embedding dimension.

Dimension	AUC	AUPR	Accuracy	F1-Score	Recall	Precision
E = 512	0.9305	0.9252	0.8609	0.8603	0.8605	0.8640
E = 600	0.9348	0.9301	0.8608	0.8607	0.8610	0.8619
E = 700	0.9360	0.9314	0.8616	0.8614	0.8618	0.8636
E = 800	0.9445	0.9422	0.8797	0.8797	0.8797	0.8789
**E = 1024**	**0.9549**	**0.9527**	**0.8907**	**0.8907**	**0.8906**	**0.8911**
E = 1200	0.9542	0.9519	0.8886	0.8885	0.8886	0.8896

Bold indicates best performance.

**Table 3:** Top 30 miRNAs predicted to be associated with breast cancer.

Rank	miRNA	Evidence	Rank	miRNA	Evidence
1	hsa-mir-21	dbDEMC	16	hsa-mir-20b	dbDEMC
2	hsa-mir-146a	dbDEMC	17	hsa-mir-145	dbDEMC
3	hsa-mir-29a	dbDEMC	18	hsa-mir-34a	dbDEMC
4	hsa-mir-222	dbDEMC	19	hsa-mir-221	miR2Disease
5	hsa-mir-196a	dbDEMC	20	hsa-mir-29b	miR2Disease
6	hsa-mir-19a	dbDEMC	21	hsa-mir-133a	dbDEMC
7	hsa-mir-19b	dbDEMC	22	hsa-mir-18a	miR2Disease
8	hsa-mir-155	dbDEMC	23	hsa-mir-146b	dbDEMC
9	hsa-mir-17	dbDEMC	24	hsa-mir-143	dbDEMC
10	hsa-mir-125b	dbDEMC	25	hsa-mir-31	dbDEMC
11	hsa-mir-126	dbDEMC	26	hsa-mir-199a	miR2Disease
12	hsa-mir-16	miR2Disease	27	hsa-mir-200c	dbDEMC
13	hsa-mir-92a	miR2Disease	28	hsa-mir-200a	dbDEMC
14	hsa-mir-15a	dbDEMC	29	hsa-mir-150	dbDEMC
15	hsa-mir-20a	dbDEMC	30	hsa-mir-9	dbDEMC

**Table 4:** Top 30 miRNAs predicted to be associated with rectal cancer.

Rank	miRNA	Evidence	Rank	miRNA	Evidence
1	hsa-mir-15a	dbDEMC	16	hsa-mir-15b	miR2Disease
2	hsa-mir-24	dbDEMC	17	hsa-mir-20b	dbDEMC
3	hsa-mir-223	dbDEMC	18	hsa-mir-193b	dbDEMC
4	hsa-mir-130b	dbDEMC	19	hsa-mir-615	dbDEMC
5	hsa-mir-140	dbDEMC	20	hsa-mir-30c	dbDEMC
6	hsa-mir-582	dbDEMC	21	hsa-mir-130b	dbDEMC
7	hsa-mir-208b	dbDEMC	22	hsa-mir-100	dbDEMC
8	hsa-mir-34a	dbDEMC	23	hsa-mir-222	dbDEMC
9	hsa-mir-16	dbDEMC	24	hsa-mir-142	dbDEMC
10	hsa-mir-145	dbDEMC	25	hsa-mir-31	dbDEMC
11	hsa-mir-29b	dbDEMC	26	hsa-mir-196a	dbDEMC
12	hsa-let-7f	miR2Disease	27	hsa-mir-199a	dbDEMC
13	hsa-mir-101	dbDEMC	28	hsa-mir-1	dbDEMC
14	hsa-let-7g	dbDEMC	29	hsa-mir-200b	dbDEMC
15	hsa-mir-221	dbDEMC	30	hsa-mir-331	dbDEMC

**Table 5:** Top 30 miRNAs predicted to be associated with lung cancer.

Rank	miRNA	Evidence	Rank	miRNA	Evidence
1	hsa-mir-29c	dbDEMC	16	hsa-mir-34a	dbDEMC
2	hsa-mir-150	dbDEMC	17	hsa-mir-125b	dbDEMC
3	hsa-mir-21	dbDEMC	18	hsa-mir-16	miR2Disease
4	hsa-mir-133a	dbDEMC	19	hsa-mir-20a	dbDEMC
5	hsa-mir-29b	dbDEMC	20	hsa-mir-222	dbDEMC
6	hsa-mir-9	dbDEMC	21	hsa-mir-15a	dbDEMC
7	hsa-mir-1	dbDEMC	22	hsa-mir-19b	dbDEMC
8	hsa-let-7e	dbDEMC	23	hsa-mir-221	dbDEMC
9	hsa-mir-199a	dbDEMC	24	hsa-mir-106b	dbDEMC
10	hsa-mir-146a	dbDEMC	25	hsa-mir-223	dbDEMC
11	hsa-mir-29a	dbDEMC	26	hsa-mir-200b	dbDEMC
12	hsa-mir-17	dbDEMC	27	hsa-mir-200c	dbDEMC
13	hsa-mir-21	dbDEMC	28	hsa-mir-19a	dbDEMC
14	hsa-mir-155	dbDEMC	29	hsa-mir-18a	dbDEMC
15	hsa-mir-145	dbDEMC	30	hsa-let-7a	dbDEMC

## Data Availability

The original contributions presented in this study are included in the article. Further inquiries can be directed to the corresponding author. The GONNMDA code is available at https://github.com/ZengsihaoNB666/GONNMDA.git (accessed on 21 October 2024).
